# Supplementary material for: Enhancing EQ-5D-5L Sensitivity in Capturing the Most Common Symptoms in Post-COVID-19 Patients: An Exploratory Cross-Sectional Study with a Focus on Fatigue, Memory/Concentration Problems and Dyspnea Dimensions
Source: Int J Environ Res Public Health. 2024 May 3;21(5):591. doi: 10.3390/ijerph21050591 (PMC11121728; doi:10.3390/ijerph21050591)

Supplementary materials

Figure S1: The prevalence of symptoms in the study population.

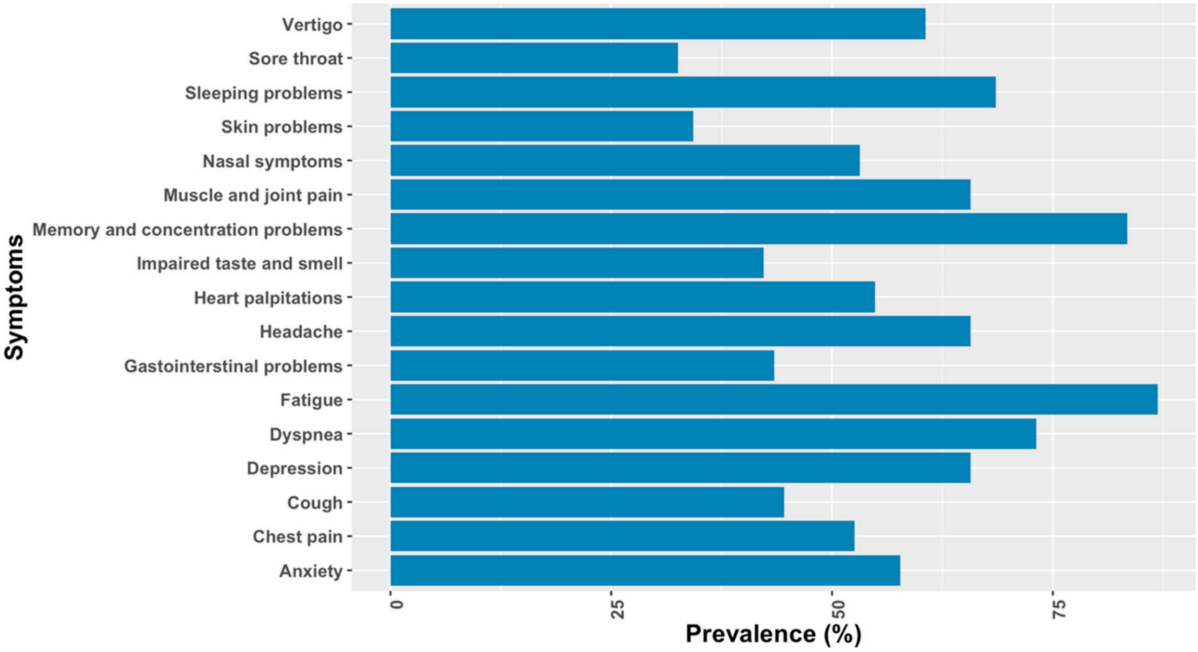

Table S1: The frequency of missing values.

Variable	Missing (n)
Country of birth	2
Education level	5
Working status	1
Working classification	42
Smoking	1
Snuff	2
BMI	5
Hospitalized	6
Symptom severity at onset	2
Cough	2
Chest pain	2

Figure S2: Variance Inflation Factor (VIF) values for EQ-5D-5L dimensions.

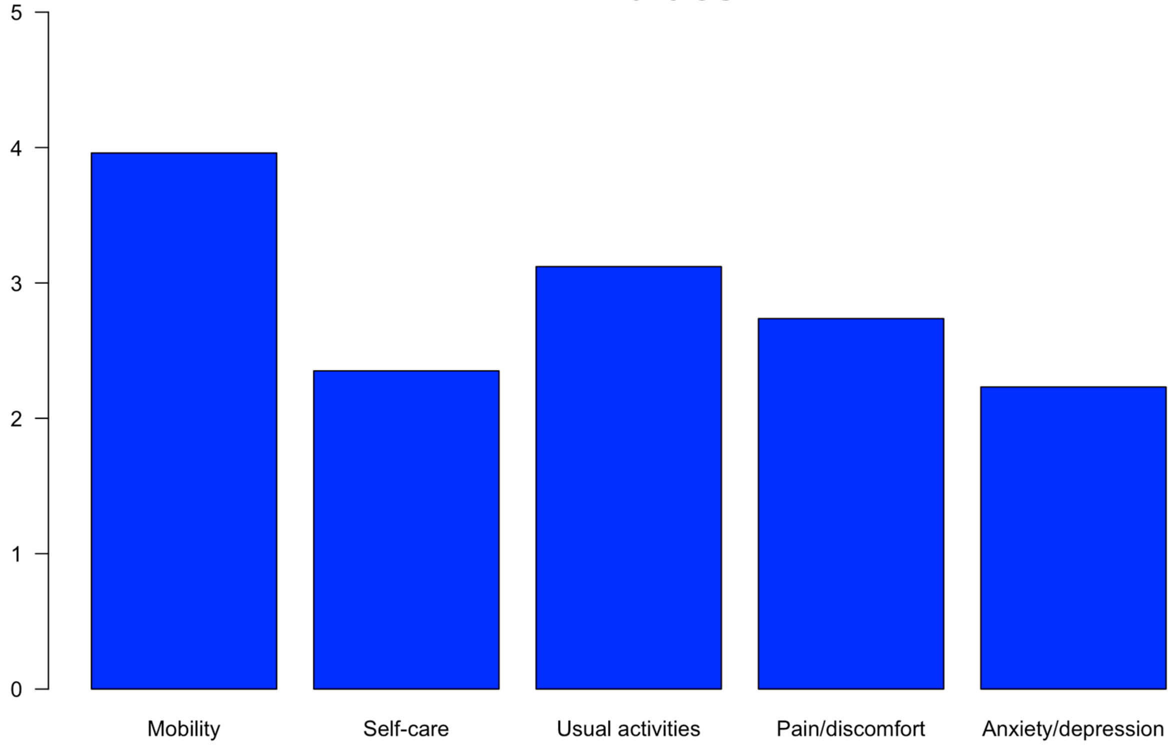

Figure S3: Heatmap of Spearman's rank correlation coefficients. The blue color represents a positive correlation and the red color represents a negative correlation between two variables. The scale to the right of the heatmap shows the strength of the correlation coefficient between the variables.

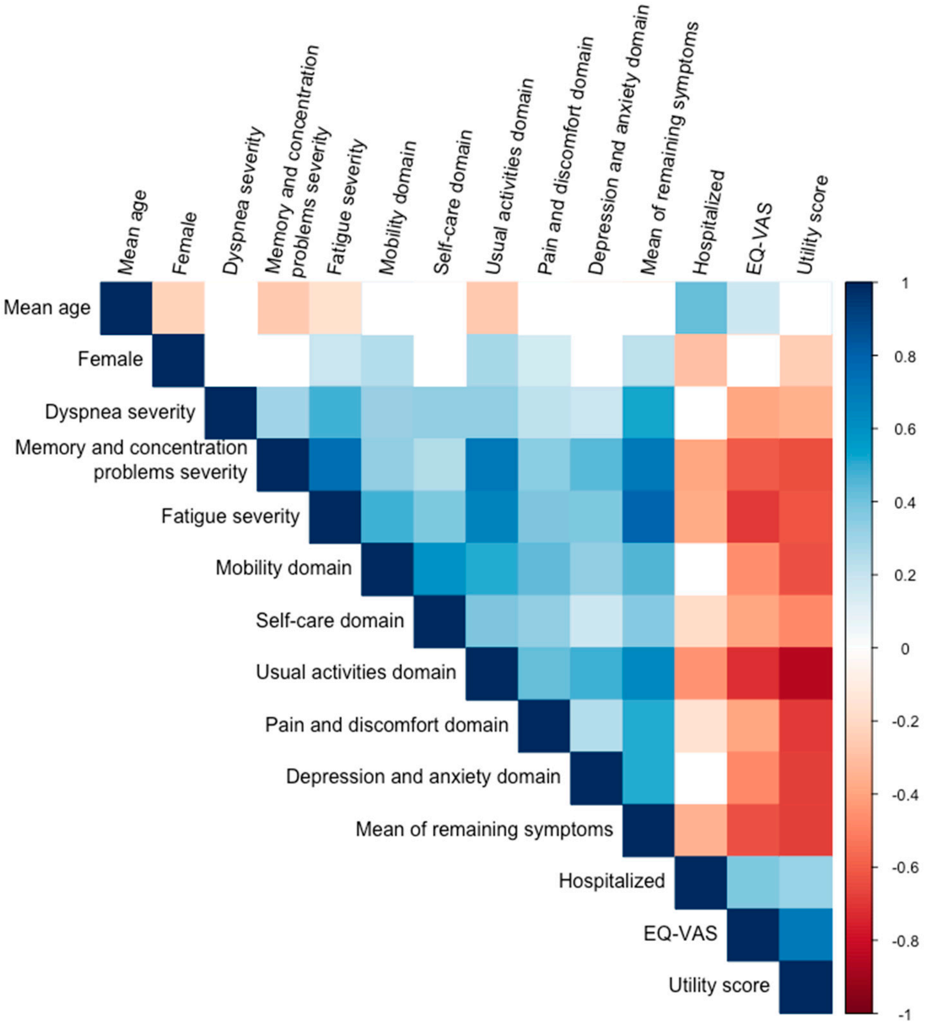

Supplement: Supplementary file 1 [file ijerph-21-00591-s001.zip › ijerph-2973552-supplementary.pdf]
